# Research progress of melatonin (MT) in improving ovarian function: a review of the current status

**DOI:** 10.18632/aging.203231

**Published:** 2021-07-06

**Authors:** Yi Ming Guo, Tie Cheng Sun, Hui Ping Wang, Xi Chen

**Affiliations:** 1 Graduate School of Peking Union Medical College, Chinese Academy of Medical Sciences, Beijing 100730, China; 2 National Engineering Research Center of Reproductive Health, National Research Institute for Family Planning, Beijing 100081, China; 3 Reproductive Medical Center, Department of Obstetrics and Gynecology, Peking University International Hospital, Beijing 102206, China; 4 Reproductive Medical Center, Department of Obstetrics and Gynecology, Peking University People’s Hospital, Beijing 100044, China

**Keywords:** melatonin (MT), ovary, follicle, reproductive system, polycystic ovary syndrome

## Abstract

Melatonin (MT) is an endogenous hormone mainly synthesized by pineal cells, which has strong endogenous effects of eliminating free radicals and resisting oxidative damages. Melatonin (MT) can not only regulate the body’s seasonal and circadian rhythms; but also delay ovarian senescence, regulate ovarian biological rhythm, promote follicles formation, and improve oocyte quality and fertilization rate. This review aimd to provide evidence concerning the synthesis and distribution, ovarian function, and role of MT in development of follicles and oocytes. Moreover, the role of MT as antioxidative, participating in biological rhythm regulation, was also reviewed. Furthermore, the effects of MT on various ovarian related diseases were analyzed, particularly for the ovarian aging and polycystic ovary syndrome (PCOS).

## INTRODUCTION

Melatonin (MT) is an endogenous indoleamine hormone secreted and synthesized mainly by the pineal gland in mammals. MT exists in the body fluid, which regulates the circadian rhythm, behavior, immune response, and reproductive function [1]. As an antioxidant, MT provides opportunities for the treatment of various diseases, including the Alzheimer's disease, cancers, immune disorders, diabetes, and viral infections [2–4].

The main function of MT is to scavenge endogenous free radicals, which can resist oxidation and prevent cellular damages. Studies have shown that MT can regulate the reproductive activities of photoperiod animals [5], biological rhythms of oocytes and ovaries, and fertilization rates. Animal studies have shown that MT can improve age-related decline in fertility and attenuate ovarian damages caused by oxidative stress [6]. The purpose of this review is to introduce the regulatory effects of MT on the ovarian physiological functions, and to illustrate the research progress of MT in the treatment of ovary-related diseases.

## MT synthesis and distribution

**Figure 1:**
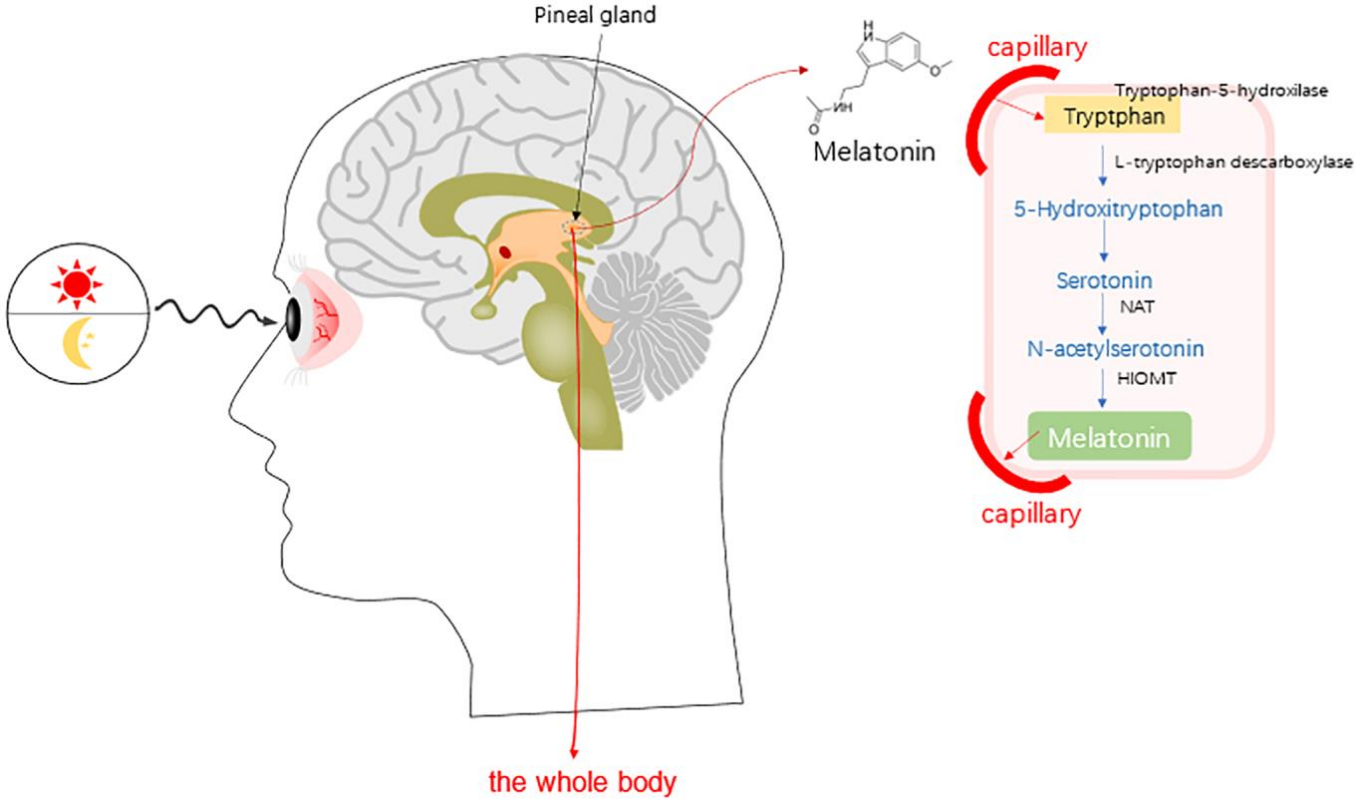
**Synthesis of melatonin (MT) in human whole body [****10****].** Abbreviations: NAT, serotonin-N-acetyl transferase; and HOMT, hydroxyindole-O-methyltransferase.

MT, with the chemical name of 5-methoxy-N-acetyltryptamine, is mainly synthesized by the pineal gland in mammals. MT can also be secreted in the retina, digestive tract, and ovary [7–9]. The pineal gland cells use tryptophan in the blood as raw materials to produce serotonin (5-HT) through the hydroxylation and decarboxylation of tryptophan hydroxylase (THP) and aromatic amino acid decarboxylase (AADC), and further synthesize MT through the acetylation and methylation of N-acetyltransferase (NAT) and oxindole-oxy-methyltransferase (HIOMT) (Figure 1) [10].

HIOMT has recently been referred to as the acetyl complex amine-O-methyltransferase (ASMT) in the human genetic database [10]. The 5-HT can be extracted from the mammal ovaries, with high expression levels of NAT and ASMT [10]. Therefore, it has been believed that the ovaries can directly synthesize MT. MT in the ovary can be derived from the systemic blood circulation, or synthesized by the granular cells, including the cumulus granulosa cells and oocytes [11]. MT can also diffuse and easily cross the morphological and physiological barriers (such as the placenta and blood-brain barrier), and then enter the cells and affect the functions of various tissues [12].

The synthesis and secretion of MT is regulated by the endogenous circadian clock in the suprachiasmatic nucleus. The levels of MT synthesis and secretion are high at night, while relatively low levels would be observed during the day, with roughly sinusoidal rhythm. The MT secretion presents a circadian rhythm of low during day and high at night, which is the basis for the physiological function of basis circadian rhythm [13].

In the process of MT biosynthesis, norepinephrine secreted by the nerve endings of the superior cervical ganglion stimulates the pineal cells through b-adrenergic receptors, thereby accelerating the synthesis of the second messenger cyclic AMP and inducing the NAT activity [14]. This pathway is actually activated at night because the nerve activity of the upper cervical ganglia would be inhibited by the stimulation of light [14]. Therefore, darkness represents the only condition for the synthesis of MT.

In human beings, the MT secretion reaches its highest level between the ages of 3 and 5 years old, which begins to decrease from the beginning of puberty [11, 14]. The secretion of MT is rather stable before the age of 35–40 years. In older age, the level of MT would be significantly diseased [15]. However, there are significant differences in the MT rhythm amplitude between individuals, and whether these differences would affect the body health has not yet been confirmed. The concentration of MT in the follicular fluid (FF) of females undergoing *in vitro* fertilization is significantly higher than in the peripheral blood [15], with circadian rhythm and seasonal changes (that is, the MT level in FF at night is higher than during day; and the MT concentrations in FF in autumn and winter with short sunshine are significantly higher than in the seasons with long sunshine such as spring and autumn) [15, 16]. During the follicles development, the MT concentrations in larger follicles are significantly higher than in small follicles, and the MT concentrations in pre-ovulation follicles are higher than that in serum, suggesting that MT plays an important role in the follicles development and ovulation [15, 16].

## MT and ovarian functions

### MT receptors in ovaries

The MT receptors could be divided into the membrane and nuclear receptors, respectively [17]. The latter is mainly related to the RZR/ROR superfamily of nuclear receptors. There are three subtypes of membrane receptors, i.e., the MT1, MT2 and MT3 receptors [17]. However, only the MT1 and MT2 receptor subtypes exist in humans and other mammals, which are encoded by the MTNR1A gene of q35·1 on chromosome 4 and the MTNR1B gene of q21-q22 on chromosome 11, respectively [18]. Based on the molecular structure, the MT1 receptor is composed of 350 amino acids, while the MT2 receptor is composed of 363 amino acids. These two receptors share 60% sequence homology, both belonging to the 7-transmembrane G protein-coupled receptor family, with similar binding site structure for MT [19].

**Figure 2:**
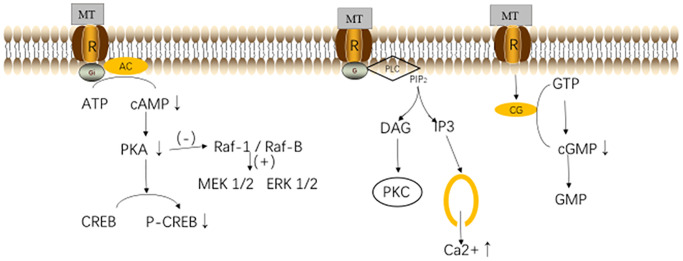
**Melatonin (MT) receptors and their pathways.** Abbreviations: MT, melatonin; R, melatonin receptor; AC, Adenylyl Cyclase; ATP, adenosine triphosphate; cAMP, cyclic adenosine monophosphate; PKA, protein kinase A; CREB, responsive element binding protein; PLC, phospholipase C; PIP2, phosphatidylinositol 4,5-bisphosphate; DAG, diacylglycerol; CG, guanylyl Cyclase; GTP, guanosine triphosphate; and GMP, guanosine monophosphate [31].

MT binds to the MT1 and MT2 receptors, which mediates a variety of physiological effects through various signal transduction pathways, including the adenylate cyclase (AC) - cAMP, mitogen-activated protein kinase (MAPK) - extracellular signal-regulated kinase (ERK), phosphatidylinositol 3 - kinase (PI-3-K) / Akt (protein kinase B), ERK-1/2 and c-Jun n-terminal Kinase (JNK)-1/2, and Akt signaling pathways [20, 21]. In different tissues and organs, MT interacts with the same receptor subtype, activating differential second messengers. The binding of MT to MT1 receptor would down-regulate the intracellular PKA activity and reduce the CREB phosphorylation, thereby inhibiting the cAMP signal transduction cascade and regulating the cell activities [22]. The activated MT1 receptor could also induce a transient increase in the concentrations of cytosolic calcium ions and inositol phosphate. In addition, studies have found that in the mouse ovaries, MT can up-regulate the intracellular AMPK signaling pathway through the MT1 receptor-mediated pathways [23, 24]. The signals mediated by MT2 receptors are related to multiple pathways (including the activation of MT2 receptors to promote the production of phosphoinositol, as well as the inhibition of adenylate cyclase and guanylate cyclase), thereby regulating the downstream pathways to exert effects [23, 24]. MT participates in the regulation of biological rhythms through a receptor-dependent mechanism, and promotes the coordination of tissues and organs [23, 24]. MT receptors exist in the human brain, cardiovascular system, liver, breast, and myometrium [17]. The mRNAs of the MT1 and MT2 receptors can be detected in human granulosa cells and luteal cells (Figure 2) [25, 26].

MT can act on the hypothalamus-pituitary-ovarian axis (HPO) by regulating the hypothalamic gonadotropins, which can also directly bind to the ovarian granulosa cells to exert effects on HPO [26, 27]. MT inhibits the expressions of gonadotropin-releasing hormone (GnRH) and GnRH receptors by up-regulating the LH receptor mRNAs [28]. GnRH, in turn, controls the secretions of the gonadotropin luteinizing hormone (LH) and follicle stimulating hormone (FSH), which regulates the reproductive function at the gonadal level and participates in maintaining the level of the corpus luteum during pregnancy [28, 29]. The wide distribution of MT receptors is the basis for its extensive biological effects. In addition, some of the biological effects of MT are receptor-independent, such as acting (as antioxidants) to prevent the oxidative stress damages [30].

### MT and antioxidation

In the ovary, oocytes and somatic cells produce the reactive oxygen species (ROS) and reactive nitrogen (RNS) in the follicular micro-environment. Active substances highly react with complex cellular molecules (such as the proteins, lipids, and DNA) and change their functions. This process would cause molecular damages, called oxidative stress. Mitochondria have been considered to be the main source of intracellular ROS and an important target for ROS supply [32]. The ROS generated by the mitochondrial respiratory chain would injure many substances, including the proteins, lipids and mitochondrial DNA (mtDNA) [32]. Cumulative damages due to excessive ROS to mtDNA may cause DNA strand breaks and lead to somatic mtDNA mutations. Somatic mtDNA mutations may cause damages to the activity of the respiratory chain complex, further aggravating the increased ROS production and mtDNA mutations [32, 33].

Oxidative stress causes the mitochondrial dysfunction and increases the uptake of Ca^2+^ by mitochondria. High concentrations of Ca^2+^ can inhibit the synthesis of ATP by mitochondria. The lack of ATP would affect cell growth and even lead to apoptosis [34]. In addition, oxidative stress can also activate the cellular apoptosis induced by caspase-3 [35]. Oxidative stress can damage the oocytes, granulosa cells and mesenchymal cells in the ovary, thereby accelerating the ovarian function failure, possibly leading to malformations and changes in the embryonic development [36, 37]. These changes would increase the cell apoptosis in pregnancy, significantly increasing the incidence of female fertility.

MT could scavenge the endogenous free radicals, which can resist the oxidation and prevent the cell damages. MT reduces the oxidative stress through various ways. It can eliminate the endogenous ROS and RNS, including the superoxide anions (O_2_^·−^), hydroxyl radicals, singlet oxygen (^1^O_2_), hydrogen peroxide (H_2_O_2_), hypochlorous acid (HOCl), nitric oxide (NO·), and peroxynitrite anion (ONOO^−^) [38–40]. In addition, MT can eliminate the metabolites formed during the interaction with oxidation products [41]. When melatonin directly neutralizes the free radicals (through the electronic donation), its derivatives are as effective as melatonin in reducing oxidative stress [42]. Melatonin and its metabolites, such as AMK, can directly scavenge the free radicals and toxic metabolites, and then form an antioxidant cascade, which plays a strong role in scavenging free radicals, directly down-regulating the intracellular ROS level, maintaining the intracellular redox balance, thus maintaining the internal environment homeostasis [43]. MT captures ROS through the 5-methoxy group on the indole ring, providing electrons to convert it into non-oxidized substances, and changing itself into a low-toxic intermediate product N1-acetyl-N2-formyl-5-methoxy canine Uracamide, which has stronger antioxidant properties than MT (removing a variety of ROS) [44]. MT is amphiphilic, which can enter into various organs and cells, thereby reducing the oxidative and nitrosative damages in the lipid and liquid environments. Therefore, compared with other classic antioxidants, MT could more effectively protect the oxidative damages [45].

**Figure 3:**
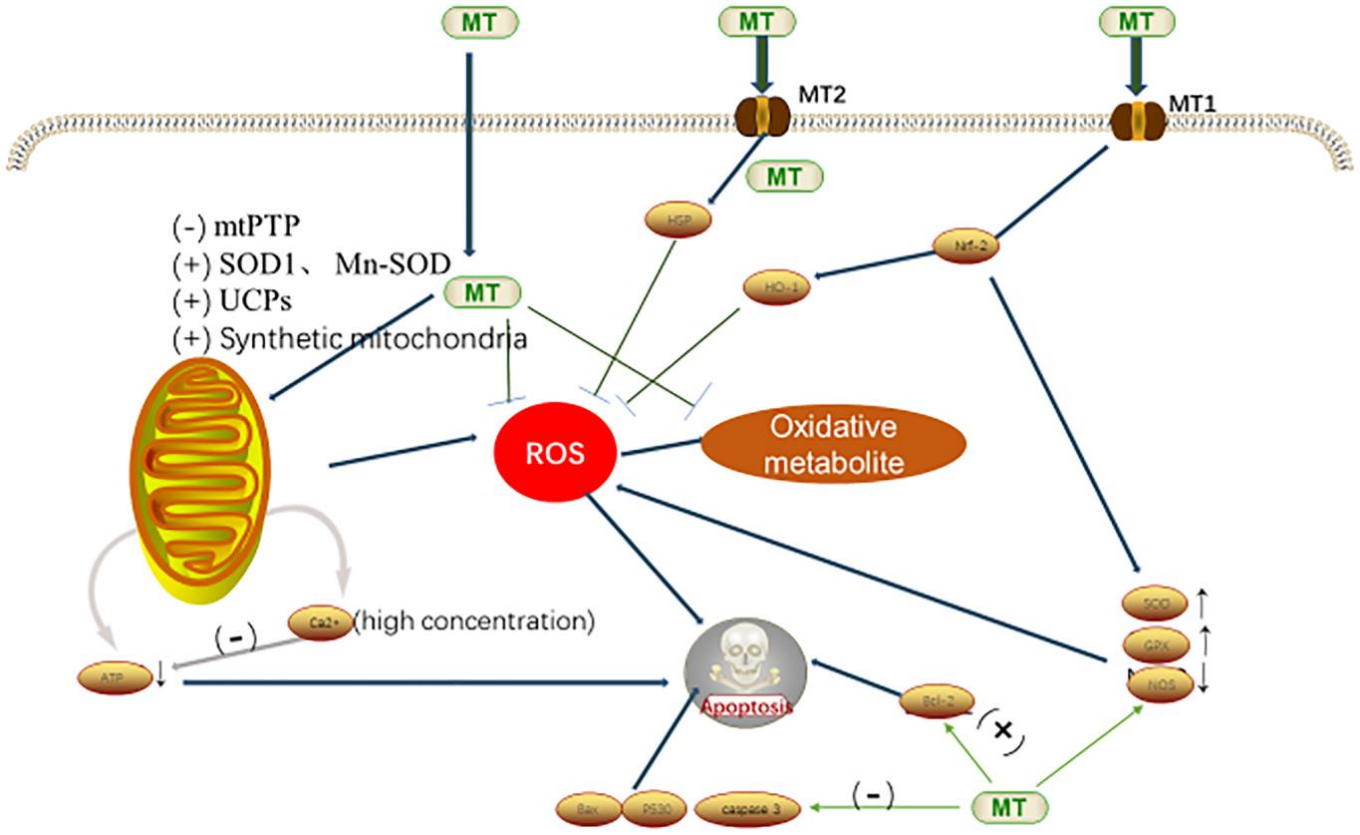
**Antioxidant mechanism of MT in cells [****7****,** **53****].** Abbreviations: MT, melatonin; ROS, reactive oxygen species; mtPTP, mitochondrial permeability transition pore; SOD, superoxide dismutase; Mn-SOD, Mn-superoxide dismutase; UCPs, uncoupling protein gene; HO-1, heme oygenase-1; Nrf-2, nuclear factor erythroid-2-related factor-2; GPx, utathione peroxidase; and NOS, nitric oxide synthase.

MT is not only a direct free radical scavenger, but also an indirect antioxidant, which can promote the expression and activity of antioxidant enzymes [i.e., the superoxide dismutase (SOD), and the glutathione peroxidase (GPX)], inhibiting the expression of the oxidative enzyme nitric oxide synthase (NOS) [41]. SIRT3-a histone deacetylases are mainly located in the mitochondrial matrix, which plays an important role in protecting these organelles from oxidative stress [46]. MT can also enhance the activity of SIRT3, activate the FoxO3a to undergo nuclear translocation, increase the binding of FoxO3a to CAT and SOD2 promoters, and lead to the transactivation of antioxidant genes, thereby limiting the production of ROS in mitochondria and inhibiting the mitochondrial oxidative damages [47]. In addition, MT reduces the expression levels of the apoptotic genes (such as the Bax, p53 and caspase-3), and increases the expression of the anti-apoptotic factor Bcl-2, thereby reducing the cellular apoptosis (Figure 3.) [7, 48].

Mitochondria are dynamic and plastic organelles that produce ATP through oxidative phosphorylation, which is key to connecting the oxidative stress and energy metabolism. MT can maintain the mitochondrial functions through the following ways: (1) by inhibiting the opening of mitochondrial permeability transition pore (mtPTP), stimulating the expression and activity of SOD1 and Mn-SOD (SOD2) in the mitochondrial matrix, regulating the mitochondrion electron flux, and reducing the mitochondrial electron leakage; (2) by increasing the activity of uncoupling proteins (UCPs), reducing the ATP production, and inhibiting the ROS production; and (3) by promoting the mitochondrial biosynthesis, and protecting the mitochondrial morphology and function [49].

Studies have shown that MT has antioxidant properties on the HPG axis [50–52]. MT can reduce the oxidative damages in the follicles, and increase the production of progesterone during the luteal phase and the maturation of oocytes [53]. Circulating MT can be absorbed by the ovaries, but the ovarian follicles have the ability to synthesize and secrete MT themselves [53]. Therefore, MT has an important paracrine effects in the female reproductive system [11, 53]. Evidence has shown that MT can scavenge the toxic free radicals, and induce the synthesis and activity of antioxidant enzymes, thus preventing the induction of mitochondrial apoptosis, and participating in the protection of granulosa cells and oocytes [7, 11, 53].

### Effects of MT on follicles and oocytes

The growth and development of follicles are rather complicated, which needs to go through the stages of primordial follicle, primary follicle, preantral follicle, antral follicle, and mature follicle. During the follicle development, oxides such as ROS and RNS would be produced. These oxides can regulate the molecular and biochemical pathways in the process of follicle formation [7, 11, 53], thus damaging the oocytes and leading to the follicular atresia. MT in human follicular fluid can attenuate oxidative stress, as well as protect oocytes and granulosa cells [37, 54]. The ROS produced by the follicles during maturation and ovulation will be eliminated by MT, while the MT is significantly reduced in the follicular fluid of elderly women [55]. When MT is used to treat infertility, it would increase the concentrations of MT in the woman follicles, thus reducing the oxidative damage sin the follicles, and improving the fertilization rate and pregnancy rate [56].

MT seems to have different functions at different stages of follicular development [56]. Some reports have shown that MT is related to the follicle stimulating hormone, which can promote the growth of goat preantral follicles and increase the production of P and androstenedione (A), in the mouse preantral follicles [21, 57]. Some studies have also described the role of MT in antral follicles, such as regulating the production of sex steroids, the expression of LH mRNA, the production of Bcl2 and Caspase3, and the activity of insulin-like growth factors and transforming the growth factor β [58, 59]. The increase in the concentration of FF MT in the growing human follicles has been considered to be an important factor in avoiding follicular atresia, because the FF MT can reduce the apoptosis of key cells, making the follicles fully developed before ovulation and providing the mature eggs for ovulation [59].

MT increases the synthesis of glutathione in human endothelial cells, and prevents the induction of mitochondrial endogenous apoptosis pathways by inducing the Bcl-2 expression and reducing the caspase-3 activity [11]. Therefore, MT protects cells from oxidative stress due to radical damages. The administration of exogenous MT can significantly reduce the oxidative damages in the follicle and the oxidative damages of essential molecules, thereby increasing the fertilization rate and pregnancy rate [11, 14]. MT regulates the responses of follicles to LH by increasing the mRNA expression levels of LH receptors in human granulosa cells [60]. In addition, MT-induced progesterone production would be produced by inhibiting CYP1 1A. CYP1 1A is a specific gene for progesterone synthesis, which increases its secretion through negative feedback, which is necessary for follicular maturation and ovulation during the dominant follicular phase [61].

**Figure 4:**
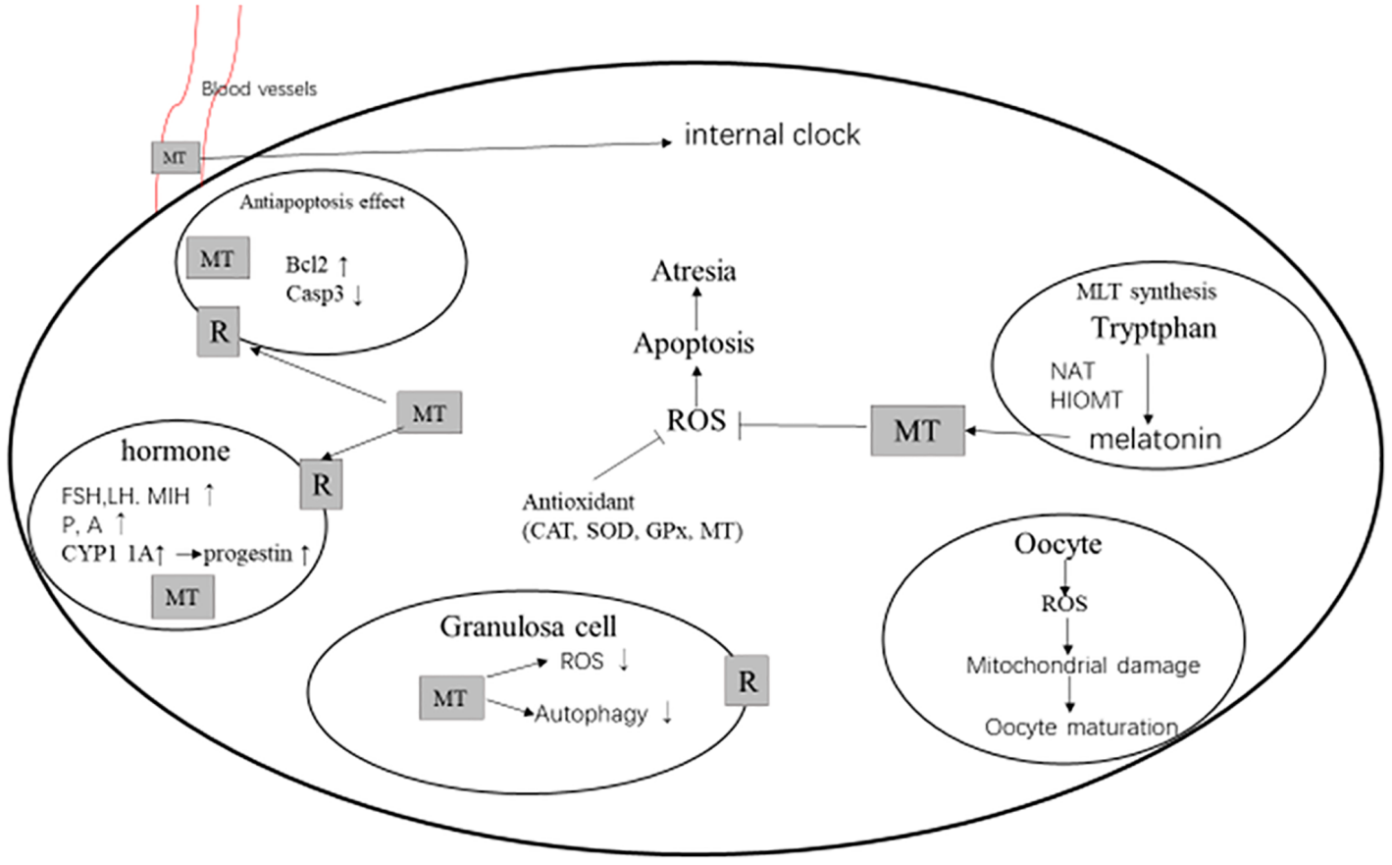
**MT and follicular development [**74**,** **75****].** Abbreviations: MT, melatonin; R, melatonin receptor; ROS, reactive oxygen species; CAT, catalase; SOD, superoxide dismutase; GPx, glutathione peroxidase; FSH, follicle stimulating hormone; LH, luteinizing hormone; MIH, maturation-inducing hormone; P, progestational hormone; A, androstenedione; NAT, N-acetyltransferase; and HOMT, hydroxyindole-O-methyltransferase.

In the process of mammalian reproduction, the maturation of oocytes is a necessary prerequisite for successful fertilization and embryo development [62]. As a powerful antioxidant, MT is beneficial to oocyte maturation and embryo development [62]. Clinically, oral administration of MT can reduce the level of oxidative stress marker 8-OHdG in the oocytes of infertile women, and increase the fertilization rate under IVF-ET treatment [56]. Studies have shown that the antioxidant function of melatonin helps to reduce the rigidity of plasma membrane, which can promote the maturation of human oocytes and early embryonic development by enhancing the reticulin mediated endocytosis (CME) [63]. MT can improve the phenotype of oocyte defects caused by maternal obesity through the SIRT3-SOD2-dependent mechanism, prevent the spindle/chromosome abnormalities in oocytes, and improve the development ability of early embryos [64]. A randomized trial has been conducted to study the application of melatonin in the assisted reproductive technology (ART), which has shown that MT can significantly improve the clinical pregnancy rate of art cycle, and increase the number of mature oocytes and the number of high-quality embryos [65]. The maturation of oocytes requires the participation of progesterone (P), which can bind to the membrane receptors of oocytes and induce the initiation of maturation-promoting factors in the cytoplasm of oocytes [66]. Under the action of the oocyte maturation promoting factor, the oocyte will undergo morphological changes, including the blastocyst rupture in the pre-deceleration division, which is a key step in the oocyte maturation [66]. Studies have shown that MT can accelerate the P action and stimulate the maturation-inducing hormone (MIH), thereby stimulating the initiation of oocyte maturation-promoting factors and the rupture of blastocysts [58, 67, 68]. Melatonin can significantly reduce the ROS levels in oocytes, improve the oxidative stress state of oocytes, reduce the early apoptosis of oocytes, repair the integrity of mitochondria, improve the spindle assembly and chromosome arrangement, and promote the meiotic maturation [69]. The MT therapy may become a beneficial treatment method to improve the ovarian function, oocyte quality and embryo development in infertile women, especially for those who cannot get pregnant due to poor oocyte quality or are about to come to the end of reproductive life (Figure 4) [70].

Other studies have shown that the combination of MT and inositol has a positive effect on oocytes [71]. In a randomized controlled trial, MT combined with inositol and vitamin D treatment can improve the quality of blastocysts and oocytes in women undergoing intracytoplasmic sperm injection (ICSI), and the study group has achieved the clinical pregnancy of 42% (vs 24% for the control group) [72]. A clinical trial has studied the effect of inositol plus folic acid and MT on the oocyte quality and pregnancy outcome *in vitro* fertilization (IVF) cycles compared with inositol plus folic acid. The study has shown that the average number of mature oocytes in the MT combination treatment group is significantly increased, and the clinical pregnancy rate and implantation rate in the combination treatment group has an increasing trend [73].

### MT regulates ovarian biological rhythm

The suprachiasmatic nucleus (SCN) of the hypothalamus stimulates the biological rhythms. It can receive light signals from the retina, and adjust the circadian rhythms of the ovary and other organs through humoral regulation and neuromodulation [75]. The biological clock system plays an important role in the physiological activities of the ovary, which is involved in the regulation of ovulation, steroid hormone synthesis and oocyte maturation [75]. Disturbance of the biological clock can seriously affect the ovarian function. SCN can regulate the secretion of MT by the pineal gland, and the local granulosa cells and oocytes of the ovary can also secrete MT [75]. There are MT receptors in the granulosa cells of the ovary, which can participate in the regulation of the ovarian clock. Studies have found that the loss-of-function mutations of the circadian clock genes Per1 and Per2 would significantly reduce the number of follicles and litter size in female mice, leading to depletion of ovarian reserve and decreased fertility [76]. Conditional knockout of the circadian clock gene Bmal1 in the ovarian steroid synthesizing cells and follicular membrane cells would result in decreased fertility and litter size in mice [77].

MT rhythm is an important outgoing hormone signal driven by an internal clock, and therefore it can be used as an internal synchronizer [78]. MT can regulate the circadian rhythm on the targets, which can also directly act on the SCN to regulate the clock [78]. Its rhythmic secretion can enhance the biological clock signal based on SCN to peripheral tissues [78, 79]. Giving exogenous MT at the same time of the day can trigger physiological and behavioral rhythms (such as body temperature and rest-activity cycles) [80]. According to previous reports, MT can regulate a variety of biological functions, including the vision, neuroendocrine, reproduction, neuroimmunity, and vascular physiology [13, 81–84]. Since the MT secretion is proportional to the night length, MT also presents a regular seasonal change cycle. MT is indeed a key parameter for photoperiod integration and induction of specific physiological responses [13].

Rhythm genes are widely present in the hypothalamic-pituitary-ovarian axis (HPO). In the cumulus-oocyte complex (COC) of rats, the expression of MT-related genes and rhythm genes could be detected. Rhythmic genes (Clock, Bmal1, Per2, and Cry1) are expressed in the rat ovary, and during oocyte maturation, the Clock, Per2, and Cry1 in cumulus-oocyte complexes (COCs) show the expression trend from high to low, along with the MLT-related genes (such as Aanat and Asmt), while the changes in the Bmal1 expression levels showing an opposite trend [85]. Removal of the pineal gland can change the expression of MT-related genes in COC, and the MT treatment can restore the expression of rhythm genes [85, 86]. These findings indicate that MT can regulate the expression of rhythm genes at different developmental stages of follicles, thereby regulating the ovarian function [68, 87]. In addition, studies have shown that MT can increase the number of eggs and high-quality embryos in patients with sleep disorders [68, 88].

### MT and ovarian aging

Ovarian senescence is characterized by gradually declined number and quality of oocytes, which may lead to infertility. Age-related decline in oocyte quality is associated with non-integral increase and immaturity of oocytes [89]. Along with the increasing age, especially after the age of 38 years, the progressive loss of human ovarian follicles will be accelerated. Although the molecular mechanism of reducing the number and quality of oocytes has not been fully understood, the process of ovarian aging is similar to the general aging mechanism [90]. Oxidative stress caused by reactive oxygen species (ROS) has been considered to candidate affecting factors for the ovarian aging, followed by the telomere length and sirtuins activity, as well as the quality of granulosa cells [55]. Telomere is a special structure located at the 3'-end of DNA, whose main function is to maintain the gene stability and protect the ends of chromosomes from DNA damages caused by ROS [55]. The regulation of telomere length is mainly achieved by telomerase. The telomerase activity and telomere length of mouse ovaries would be decreased along with reproductive aging [91]. The expression of sirtuins (SIRT1, SIRT3, and SIRT6) in the ovary is positively correlated with ovarian reserve [92]. These proteins may be potential markers for ovarian aging, and target molecules for delaying the organ aging SIRT1, SIRT3, and SIRT6. Studies have shown that long-term application of MT can delay the ovarian aging [6, 55]. A previous study has reported that the long-term administration of MT to Kunming mice can significantly reduce ovarian aging, as manifested by significantly increased numbers of fetuses, follicular pools, telomere length, as well as the number and quality of oocytes [6].

MT treatment significantly prolongs the telomere length of aged mice, which may attenuate the age-related telomere shortening in oocytes [55]. The MT treatment can activate the ovarian SIRT1 and SIRT3 mRNA expression levels. Studies have shown that in mice and human beings, the up-regulated expression of SIRT1 induced by MT is related to the reduced oxidative stress, activated antioxidant enzymes, and anti-apoptosis effects [93, 94]. MT delays the senescence of mouse oocytes after ovulation through the SIRT1-MnSOD-dependent pathway [95]. MT also inhibits the autophagic death of hepatocytes induced by cadmium by enhancing the activities of human SIRT3 and superoxide dismutase 2 (SOD2) [69]. Therefore, MT may protect ovarian cells and reduce follicular atresia by activating SIRT1 and SIRT3 signals.

Ribosome-related genes are up-regulated in the ovaries treated with MT, indicating that MT may regulate its function by participating in the translation process of ribosomes [69]. Accurate protein translation and normal ribosomal function are crucial in delaying cell senescence [96]. MT acts directly as an antioxidants, scavenging free radicals, and delaying the decline of oocyte quantity and quality [69]. MT can also indirectly enhance the activity of antioxidant enzymes, or reduce the oxidative stress of the ovary by regulating the mitochondrial function. In addition to the above pathways, MT may also depend on the receptor-mediated binding to MT1 and MT2 receptors, mediating multiple physiological effects through various signal transduction pathways, to fight against premature ovarian failure [6, 91].

### MT and polycystic ovary syndrome (PCOS)

Polycystic ovary syndrome (PCOS) is a common endocrine disease in women of childbearing age. The clinical symptoms are mainly manifested in anovulatory infertility, hyperandrogenism and polycystic ovarian diseases. The incidence rate has been gradually increased in recent years, up to 6%-10% [97]. PCOS is a multi-gene-related disease, which is characterized by a complex genetic pattern, including the hyperandrogenism, ovulation dysfunction and polycystic ovary changes [98]. In addition, the endocrine and metabolic abnormalities of PCOS patients are also manifested as the increased serum luteinizing hormone (LH), decreased follicle stimulating hormone (FSH), increased serum androgen level, hyperinsulinemia, insulin resistance (IR), obesity and dyslipidemia. The risks of hypertension and cardiovascular diseases would be significantly increased [98].

Studies have shown that the MT levels in serum and saliva of PCOS patients are higher than healthy women, and the levels of urinary-6-hydroxysulfated melatonin (aMT6s), an important metabolite of MT, are also significantly increased, while the MT level in follicular fluid is on the contrary [74, 99]. The MT level in mature follicles before ovulation is much higher than the immature follicles [100]. Therefore, it is suggested that the decreased MT level in follicular fluid of PCOS patients is due to decreased absorption and excessive follicular atresia [75].

MT has obvious influence on the clinical, endocrine and metabolic characteristics of PCOS patients. MT can inhibit androgen-estrogen conversion in granular cells, leading to decreased estrogen levels, thereby causing negative feedback release from the hypothalamus-pituitary axis, and increasing the FSH secretion through pituitary stimulation [101]. A study has shown that after 6 months of MT treatment, the androgen level and anti Mullerian hormone (AMH) serum level of PCOS patients are significantly decreased, while the FSH level is significantly increased, and the parameters of blood glucose and blood lipid (except low density lipoprotein) have no significant change [102]. A randomized, double-blind, placebo-controlled clinical trial has been conducted in 56 patients with PCOS, and the results show that MT significantly reduces hirsutism, serum total testosterone, high-sensitivity C-reactive protein (hs CRP), plasma malondialdehyde (MDA) levels, and significantly increases the plasma levels of total antioxidant capacity (TAC) and total glutathione (GSH) [103]. MT supplementation can significantly reduce the Pittsburgh sleep quality index, Beck Depression scale index (BDI) and Beck Anxiety Scale index, which can significantly reduce the serum insulin, homeostasis model of insulin resistance (HOMA-IR), serum total cholesterol and LDL cholesterol levels, and significantly improve the quantitative insulin sensitivity test index (QUICKI) [104]. In addition, inhibiting aromatase activity at the ovarian level can increase androgens in the ovaries, thereby improving the follicular sensitivity. MT could also exert the effects and functions, as the selective estrogen enzyme modulator (SEEM) and selective estrogen receptor modulator (SERM) [99].

PCOS leads to anovulation in women of childbearing age, which is one of the pathological factors of *in vitro* fertilization (IVF) failure [105]. It is very important for PCOS patients to improve the quality of oocytes. It has been shown that, MT supplementation (3mg/day) can help improve the pregnancy rate by reducing the concentration of 8-hydroxy-2'-deoxyguanosine during IVF [56]. A double-blind randomized clinical trial has been conducted in 198 PCOS patients undergoing intrauterine insemination (IUI), which indicates that the MT (3mg/ml) treatment can improve the quality of follicles and significantly increase the chemical pregnancy rate [106]. MT promotes the expression levels of CYP19A1 and HO-1 in human ovarian GCS, reduces the level of IL-18, and promotes the oocyte maturation in PCOS patients with hyperandrogenemia [107]. In addition, MT has been proved to effectively stimulate the nuclear maturation of oocytes and improve the maturation rate by regulating free radicals to a certain level [37]. In a randomized double-blind trial of PCOS patients, MT and inositol can synergistically improve the ovarian response to gonadotropin stimulation at the ovarian level, thereby improving the quality of oocytes and embryos [108].

The application of conventional treatment to control ovarian hyperstimulation in patients with PCOS would lead to a higher risk of ovarian hyperstimulation syndrome (OHSS). Insulin resistance of ovarian granulosa cells and over-expression of vascular endothelial growth factor (VEGF) caused by insulin stimulation have been considered to be potential mechanisms underlying the adverse clinical outcomes [109]. The combination of MT supplement and exercise behavior may (through up-regulating GLUT4 and PGC-1α and mitochondrial biogenesis mechanism) improve antioxidant activity, hyperlipidemia, and inflammatory cytokines, thereby improving IR [110].

Menstrual cycle disorder is one of the main complications of PCOS, which seriously affects the patients’ quality of life. The disorders of HPO axis in PCOS patients lead to the disorder of follicle maturation and ovulation, while IR and hyperinsulinemia also lead to ovarian dysfunction, leading to anovulation and menstrual cycle disorder in PCOS. One study has investigated 40 PCOS patients who took MT for 6 months, and 95% of them had improved menstrual cycle [102]. So far, however, there are few data about MT improving the menstrual cycle of PCOS patients.

## Summary

A large number of studies on mammalian MT have made great progress in understanding the MT action mechanism on ovarian function (Table 1). There is evidence that MT acts through a variety of receptors, or it can be directly used as a direct free radical scavenger without receptor action. A large number of experimental data show that MT can participate in the ovarian physiology, including the follicular development, ovulation, oocyte maturation, and ovarian biological rhythm. The lack of MT is one of the causes of ovarian aging, polycystic ovary syndrome and other diseases. In clinical application, MT function has been explored and applied. Even if the application of MT is limited, it provides a good basis for future exploration.

**Table 1:** References concerning melatonin (MT) improving ovarian function.

	**Animal/people**	**Design**	**Melatonin regulation results**	**Year**	**Author/Reference**
Follicle	Sheep	*vitro*, IVM	The lowest MT level was seen in the small follicles, but there was no significant difference between medium follicles and large follicles AANAT, HIOMT, MT1, and MT2 mRNA expression levels in COCs were decreased with increasing follicle diameter	2012	Xiao L [21]
Oocyte	Carps	*vitro*, IVM	GVBD↑, the histone H1 kinase activity in oocyte↑, acceleration in histone H1 Phosphorylation↑	2004	Chattoraj A [65]
Oocyte	Mouse	*vitro*, IVM, implantation	Gss, Atp6, Atp 8, Tet1, Tet2, and Tet3 mRNA expression↑ normal distribution rates of IP3R1, CD9 protein, mitochondrial, CGs and ER, Juno expression↑ the representative images of DNA methylation analysis of promoters of CD9 and Juno genes↑ two-pronuclear embryos rate, cleavage and blastocyst rates↑ Dnmt1↓, DNMT1↓, TET1↑, the normal distribution rates of↑	2018	Zhao XM [68]
Oocyte	mouse	*vitro*, IVM	proportion of PBE in BPA-exposed oocytes↑ did not affect GVBD a majority of disorganized spindle morphologies and misaligned chromosomes was observed in BPA-exposed oocytes restores the defective kinetochore-microtubule attachment rates of fertilization, sperm binding ability↑ ZP2 cleavage level, rescues localization and protein level of ovastacin↑ June↑, ROS↓		Zhang M [69]
Biorhythm	Mouse	*vitro*, IVM	clock genes (Clock, Bmal1, Cry1, Cry2, Per1, Per2) in COCs↑	2015	Coelho LA [77]
Ovarian senescence	Mouse	*vitro*, IVM	SIRT1, Bcl2↑, ROS↓	2015	Yang Y [111]
Ovarian senescence	Mouse	*vitro*, IVM	Counteracted age-related fertility decline, oocytes↑, ovarian mitochondrial oxidative stress and apoptosis↓	2016	Song C [6]
Ovarian senescence	Mouse	*vitro*, IVM	SOD, CAT, MDA, SIRT1, Ac-FoxO1, Ac-p53, Ac-NF-κB, Bcl-2↑, Ac-FoxO1, Ac-p53, Ac-NF-κB, Bax↓	2015	Zhao L [93]
PCOS	people	clinic	LH, FSH, testosterone↑	2004	Luboshitzky R [102]
PCOS	people	clinic	MT levels were found to be positively associated with increased testosterone	2013	Jain P [97]
PCOS	people	randomized controlled trial, *vitro*	the effect of myo-inositol and MT improving *in vitro* fertilization of patient with PCOS is better than myo-inositol	2015	Pacchiarotti A [109]
PCOS	mouse	*vitro*, IVM	LH, FSH, IVM↑ lower doses MT enhanced maturation rate	2017	Nikmard F [108]
PCOS	people	clinic	MT treatment can restore menstrual cyclicity in women with PCOS	2018	Tagliaferri V [103]
PCOS	people	clinic	The level of MT in serum and saliva of PCOS patients was higher than that of healthy women	2013	Jain P [97]
PCOS	people	clinic	MT in urine of patients with PCOS, aMT6s↑	2001	Luboshitzky R [99]
PCOS	people	clinic	Androgen, AMH↓; FSH↑, blood glucose and blood lipid had no significant change; The menstrual cycle was improved in 95% of the subjects	2018	Tagliaferri V [103]
PCOS	people	clinic	Hirsutism, serum total testosterone, hs CRP, MDA↓ TAC/GSH↑	2019	Jamilian M [104]
PCOS	people	clinic	Pittsburgh sleep quality index, Beck Depression scale index and Beck Anxiety Scale index; QUICKI↑	2019	Shabani A [105]
PCOS	people	clinic	Supplementation of 3 mg/day melatonin can help improve the pregnancy rate by reducing the concentration of 8-hydroxy-2′- deoxyguanosine during IVF	2019	Tamura H [55]
PCOS	people	clinic	Given 3 mg/ml melatonin treatment, can improve the quality of follicles, improve the pregnancy rate	2019	Mokhtari F [107]
PCOS	people	clinic	The expression of CYP19A1 and HO-1 in GCs was↑; IL-18↓; Promote the decrease of IL-18 level, promote the oocyte maturation of PCOS patients with hyperandrogenemia	2019	Yu K [37]
PCOS	people	clinic	GLUT4, PGC-1 α↑; Improve mitochondrial mechanism, improve antioxidant activity, hyperlipidemia, inflammatory cytokines	2017	Rahman MM [112]

Abbreviations: AANAT: arylalkylamine N-acetyltransferase; HIOMT: hydroxyindole-O-methyltransferase; COCs: cumulus–oocyte complexes; E2: β-estradiol; A: androstenedione; P: progestin; MIH: maturation inducing hormone; GVBD: germinal vesicle breakdown; CGs: cortical granules; ER: endoplasmic reticulum; Cat, Sod1, GPx: endogenous antioxidant genes, IP3R1 distribution and expression of CD9 and Juno: fertilization-related events, BPA (Bisphenol A), PBE: Polar Body Extrusion; GVBD: Germinal Vesicle Breakdown); Hs CRP: high sensitivity C-reactive protein; MDA: plasma malondialdehyde, TAC: plasma total antioxidant capacity; GSH: total glutathione; QUICKI: quantitative insulin sensitivity index.
